# Village mentoring and hive learning: The MIT Critical Data experience

**DOI:** 10.1016/j.isci.2021.102656

**Published:** 2021-06-10

**Authors:** Christopher V. Cosgriff, Marie Charpignon, Dana Moukheiber, Mary E. Lough, Judy Gichoya, David J. Stone, Leo Anthony Celi

**Affiliations:** 1 Department of Medicine, Hospital of the University of Pennsylvania, Philadelphia, PA 19104, USA; 2 MIT Institute for Data, Systems, and Society, Cambridge, MA 02138, USA; 3 University at Buffalo, Buffalo, NY 14260, USA; 4 Primary Care and Population Health, Stanford University, Stanford, CA 94305, USA; 5 Emory University, Atlanta, GA 30322, USA; 6 Departments of Anesthesiology and Neurosurgery, and the Center for Advanced Medical Analytics, University of Virginia, Charlottesville, VA 22908, USA; 7 Laboratory for Computational Physiology, Massachusetts Institute of Technology, Cambridge, MA 02138, USA; 8 Department of Medicine, Beth Israel Deaconess Medical Center, Boston, MA 02215

**Figure:**
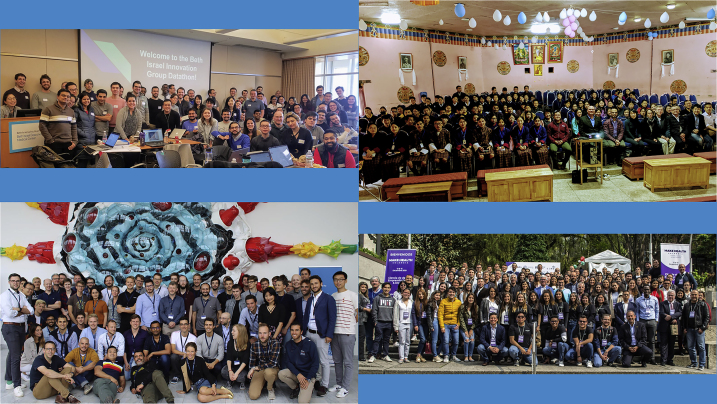
The scientists who contributed to this backstory are but a very small fraction of the MIT Critical Data consortium. The group expands with every datathon that is organized. The first photo was taken at Beth Israel Deaconess Medical Center in Boston (top left); the second was at Khesar Gyalpo University of Medical Sciences in Thimpu, Bhutan (top right); the third was at Universidad de los Andes in Bogota, Colombia (bottom right); and the last was at Aarhus Universitet in Aarhus, Denmark (bottom left). These events are organized with a local university and/or hospital.

A lot of what we're doing is connecting people and aligning incentives. In order to advance, hospitals need to work with universities who need to work with industry. Industry partners need access to clinicians and data.

Therefore, there has to be a component of project management and recognition and direction to avoid feeling insecure in projects that are very technically intense.

This is an excellent question as work can be too technical for clinical journals, and too clinical for technical journals. It can even be hard for journals to obtain reviewers who are capable of reviewing all the components of these works. However

Our tip would be to keep an open mind and to continue to ask questions and not to be afraid to ask what might appear to be “stupid” questions: “Stupid” questions in one area may be fundamentally important in another.

It is often said that every system is perfectly designed to get the results it achieves. This invocation challenges us to rethink the systems we have in order to produce the results that we want. Consider then our current biomedical research apparatus and how its training and funding processes foster the crisis of replication, the insular anti-collaborative nature of closed data, and a system that preserves existing power structures and rewards narcissism. The COVID-19 pandemic has further highlighted many of the weaknesses of our current approach. A key feature of the current system is the structure of research groups which may be conceptualized as mentorship hierarchies led by principal investigators. While intergroup collaboration is common, the structure rewards competition between groups and incentivizes selfishness: trainees developing within this structure are compelled to become hyperspecialized in hopes of obtaining grant funding in a unique area and developing into principal investigators with groups of their own.

Over the past decade, MIT Critical Data has been emblematic of a different approach: the Village of Mentors. Founded initially around the goal of democratizing the use of electronic health record data to advance observational research and inform care, MIT Critical Data has evolved a broader paradigm for collaborative research that emphasizes intergroup collaboration wherein members are simultaneously mentors and mentees in real time depending on a project's context and requirements. Spanning the globe and disciplines they have worked together on countless projects leading to numerous publications and the proliferation of novel open source health databases. In this backstory the founder of MIT Critical Data, Leo Anthony Celi, and various members of the group discuss their experience in establishing and occupying the Village of Mentors.

## Proximity

### What was the motivation behind MIT critical data and village of mentors as an interdisciplinary education approach?

**Christopher V. Cosgriff (Hospital of the University of Pennsylvania):** “If you build it, they will come.” MIT Critical Data grew organically out of a belief the current biomedical research apparatus is insufficient in its efforts to solve the panoply of health issues our society faces today. Understanding that every minute that health data sits in a silo is another lost opportunity to advance our understanding of disease, the Laboratory for Computational Physiology developed an open-access clinical database containing all of the electronic health record data collected in of adult intensive care units (ICUs) at the Beth Israel Deaconess Hospital and entitled it MIMIC (“Medical Information Mart for Intensive Care”).

Data hackathons (“datathons”) were held to promote access and use and to provide a forum for education and collaboration. The interest in such events spread like wildfire, and soon they were being led across the world. We realized quickly that by bringing clinicians and data scientists together we were breaking down a barrier and opening the floodgates. The groups that formed persisted and continued to work together. Buttressed by open-access code repositories, groups formally and informally collaborated to solve shared problems as they sought to answer different questions; everybody learned.

**David J. Stone (University of Virginia):** The motivation was based on necessity-the issues that MIT Critical Data addresses cannot be fully addressed by any single discipline. Besides the simple physical challenges of getting clinicians and data scientists (among others) face to face, there are fundamental linguistic and cultural differences between doctors and the other involved technical specialties that can make communication and collaboration difficult. For example, doctors often work in an iterative manner, making decisions and interventions, observing the results, and then adjusting (or not) their approach to the clinical problem. On the other hand, computer engineers are much more likely to plan everything in advance and carry out their entire process without such frequent re-iterative reanalyzes.

**Leo Anthony Celi (MIT Laboratory for Computational Physiology):** A lot of what we are doing is connecting people and aligning incentives. In order to advance, hospitals need to work with universities who need to work with industry. Industry partners need access to clinicians and data. With each stakeholder lacking these resources we met a valuable need and created a space for these varied stakeholders to come together.

### What are the main challenges you faced so far and your projects for the future?

**Marie Charpignon (MIT Institute for Data, Systems, and Society):** Access to electronic health record (EHR) data across countries remains challenging: despite successful partnerships with South Korea, the Netherlands etc., one challenge is for the Critical Data Lab to encourage the broader adoption of best practices for clinical data standardization, e.g., by explaining the benefits of common data schemas such as the one adopted by the large open critical care databases MIMIC-III and MIMIC-IV and by diffusing them more broadly. The field of health data science would gain tremendously from more interoperability between EHR data sources.

Going forward, more shadowing opportunities could be proposed for data scientists to familiarize with the daily challenges that clinicians face and for doctors to discover the complexities of retrospective data processing and analysis when the information is taken out of the clinical context. Additionally, more fellowship opportunities should be offered for the future generation of health data scientists to successfully engage with partners across the spectrum: software providers, hospitals, biostatisticians, lab-based researchers, and regulatory institutions.

## Sharing

### What are the key factors that stimulate interdisciplinary research, and how are these applied in the village of mentors?

**Judy Gichoya (Emory University):** Trust – Interdisciplinary work requires having a “connection” with someone, usually who is influential in the specific network you are trying to penetrate. The Village of Mentors relies on sharing trust as a privilege. Therefore, there are always new introductions to members of the village and external collaborators, shortening the time it takes to get to know people and work together.

Open collaboration – There needs to be a flow of ideas, and hence some form of regular meetings and check-ins need to occur. They do not need to be the whole group, but the members of the Village are always interfacing with each other and interacting with each other … and, hence expanding their connections. There needs to be infrastructure that supports and lowers the bar for participation. MIMIC has lots of tooling and in the village has the data experts. Therefore, blockers to data centric projects are easily overcome as experts are easily reached.

Sense of equity in shared opportunities or “being able to see the pie that it is big enough for everyone” – the academic currency is publications – specifically on the position of the authors – first or last. There is an equitable distribution of academic credit in the village of mentors – usually achieved by modular design of projects so that multiple papers and presentations arise from the joint projects/research. Because of the influx of ideas and multiple experiments, there are always parallel smaller projects from ideas that arise during discussions. Usually, the decision on authorship is NOT the first item discussed in this model.

Diversity – in ideas, experience, and opinions. There are no “permanent members of the village” but people are unified by the needs required in a project. For example, in a project where we are studying bias in this mentorship model, the team has expanded because of the technical and academic challenges demanded by the project. We are always looking internally at what and who is missing for us to have a complete picture of the project at hand.

**Charpignon:** Students, mentors, clinicians, and professors engaged in the network all share a genuine interest both in improved and more equitable delivery of health-care services and see the value of the large-scale collaborative data science in medicine. With humility, they are willing to share their expertise with team members from other fields during workshop days with the objective of reinforcing interdisciplinarity and training the next generation of health data scientists. For example, a causal inference specialist would run a session on mediation analysis, including hands-on sessions with demos where members would experiment statistical tooling and software at their own pace, while a pneumologist and critical care specialist might give a tutorial on the different types of mechanical ventilation and how to adapt respiratory therapy to each patient's profile in ICU settings.

## Language

### How does this approach help prepare students and mentors to communicate in an interdisciplinary setting and to solve conflicts?

**Stone:** Interdisciplinary communication starts with respect for the knowledge and ability of other specialists, and then builds on activities such as datathons that educate all sides regarding domains with which they are not traditionally familiar. Mentors start with an understanding of this context and function as interfaces facilitating interactions. Conflicts are generally resolved by gaining a greater understanding of the other side's viewpoint, and this approach is not only encouraged but required to participate.

**Celi:** Our strategy has been to seek out a network of collaborative individuals with varying expertise; in all honesty, there is a lot of self-selection by way of the datathon. As Judy said earlier, trust is the foundation of how we work together. Sometimes people try to hide conflict under the umbrella of academic disagreement, but it is usually egos that are clashing. The secret is creating an environment that is safe. It is basically what we do in medical training. You pre-emptively avoid conflicts by creating an environment where there is trust and people are safe.

A lot of it is about being a role model. I mean this is not rocket science. You have to invest in your people. At MIT Critical Data, we are connoisseurs of talent and it all comes down to culture. Our governance is ultimately about role modeling. We created a culture that emphasizes “how do I contribute to the learning of the group” rather than “how does this contribute to my career.” It is hard to turn that into a governance structure, it is more of an ethos than a set of rules.

**Charpignon:** The Critical Data Lab is fundamentally participatory: even early in their research career, team members are encouraged to engage with research communication and writing, learning from their senior peers about methods to conduct a literature review and nuance the study results. Team members in doctoral training are also invited to participate in the drafting of grant proposals. This is another active learning step, often critical for the generation and framing of future research ideas.

Interdisciplinary and intergenerational teaming is also key to student training. For those in the Greater Boston Area who can participate in-person to our courses and workshops, interactions are favored between undergraduate and graduate students as well as residents and established doctors, across a wide range of specialties and across generations. Through a semester-long project, teams of four to six members learn about data collection and pre-processing, investigate potential biases in the data, and develop modeling and visualization tools, paying specific attention to clinical interpretability, science communication, and feasibility of deployment in hospital settings or at the population-level.

## Challenges

### What challenges can be faced by interdisciplinary researchers and how does MIT critical data help prepare students and researchers for them?

**Gichoya:** Interdisciplinary work requires fit and chemistry between researchers and students. Therefore, you must understand what the core pillars of the project are. Moreover, for technical projects, some of the members can be challenged by high technical requirements e.g. requiring programming skills for medical professionals hence intimidation or development of egos. It is necessary to identify what the core strengths of the members are, will they source data sets? Will they supervise students? Therefore, there has to be a component of project management and recognition and direction to avoid feeling insecure in projects that are very technically intense. It is worth noting that as the US government is more critical of foreign influence on research – collaboration and funding – it will be interesting to see the impact of this on international collaborations.

**Charpignon:** The field of machine learning for health makes progress at an incredible pace. In such context, the team strongly believes in the benefits of continued education for all. Beyond the combination of in-person and online learning sessions, the team has invested in open-access education early on, leveraging platforms such as edX. Since the Critical Data lab was created, hundreds of videos, quizzes, exercises, and case studies in medical informatics and public health data science have been co-created by mentors and released to the e-learning community in open access (https://www.edx.org/course/collaborative-data-science-for-healthcare; https://www.edx.org/course/global-health-informatics-to-improve-quality-of-ca), benefiting students worldwide. In addition, and thanks to the feedback shared by students who participated in such online or hybrid learning programs, the team has co-authored two books – now edited in several languages – that set the foundation of each course and contribute to the large diffusion of the training in health, sciences, and technology offered at MIT and Harvard.

## Publication

### When publishing interdisciplinary work, what strategies have you applied in reaching a broader audience beyond specific disciplines?

**Stone:** This is an excellent question as work can be too technical for clinical journals, and too clinical for technical journals. It can even be hard for journals to obtain reviewers who are capable of reviewing all the components of these works. However, the publishing world has gradually evolved to accommodate such work with journals such as Nature Digital Medicine, Lancet Digital Health, the new PLOS digital health, BMJ Health and Care Informatics all augmenting the traditional medical informatics journals. Plus journals such as JAMA are now publishing more digitally oriented research as are medical specialty journals such as Intensive Care Medicine and Critical Care Medicine.

**Charpignon:** Over the years, the Village of Mentors has built upon open science and seamless collaboration frameworks. Most of the code underlying the MIMIC-III, MIMIC-IV, and eICU critical care databases that the group has made broadly available is available on Github, with the possibility for users to submit questions and revisions at any time. Most members of the Critical Data lab use Slack on a regular basis to communicate and foster collaborations that started in class or during datathon events around the world. These sustained connections contribute to the attractivity of MIT Health Sciences & Technology department, since visiting students from Milano, Paris, Singapore, São Paulo, and many other places where the team held events often come to the lab for an exchange semester or a research gap year.

## Future

### What tips would you give to anyone considering undertaking interdisciplinary work?

**Dana Moukheiber (SUNY Buffalo):** I would encourage anyone interested in applying their data science skills to the health-care domain to get involved in interdisciplinary research and datathons organized by MIT Critical Data around the world and open broadly to the clinical, engineering, and data science communities. It is a great way to stay up to date with recent breakthroughs in health care while being part of a Village of Mentors — a community comprising individuals with diversity of expertise and extensive domain knowledge contributing to solve meaningful world problems. It takes a team to oversee a project all the way from developing strong data governance policies, to investing in necessary computational resources, to pre-processing data, to developing models, to continuously monitoring the model performance as new data comes in from prospective or retrospective studies and finally to deploying the model to production. Assembling interdisciplinary agile teams will get us closer to the adoption of responsible AI in health-care systems.

**Stone:** The future is bright as it seems that digital medicine, including artificial intelligence approaches, will become progressively more important components of the health-care system. Our tip would be to keep an open mind and to continue to ask questions and not to be afraid to ask what might appear to be stupid questions: Stupid questions in one area may be fundamentally important in another.

